# Artificial Dilatation of the Cervix in Obstinate Vomiting of Pregnancy

**Published:** 1876-01

**Authors:** 


One other point is worthy of consideration, viz: Cold is taken just as easily by the rapid passage from a very cold, or damp atmosphere, to one where the temperature is excessively dry or elevated, as by the sudden change from the latter conditions to those first mentioned. We should be careful therefore to counsel nurses—

1st. Not to keep the nursery at a too high temperature, and always to have a basin, pitcher, or bucket full of fresh, pure water, with the surface exposed to the air contained in the room.

2d. Not to allow children to approach a fire-place or register immediately upon entering or leaving the house.

3d. Not to keep extra wraps on their little charges, when at home, a moment longer than is absolutely essential.

Practical attention to the preceding rules, and also to what we have written with respect to cold bathing, care of the feet, etc., as prophylactic measures of hypertonsillar growth, will be found, we are firmly convinced, of unquestionable utility.

Artificial Dilitation of the Cervix in the Obstinate Vomiting of Pregnancy.—In the proceedings of the Obstetrical Society of Boston (*Boston Medical and Surg. Journal*) Dr. Sinclair referred to cases recently reported by Dr. Copeman. Dr. Copeman was called to a patient six months advanced in pregnancy. In endeavoring to break the membranes, he failed after two or three efforts. He retired for an hour to consult, and in the mean time the vomiting had ceased. The patient did well without further interference. In another case, a pregnancy of two months, abortion was proposed, and the cervix was dilated with the same result. In another patient, eight months along, a like effect resulted from the same treatment. Graily Hewitt, in commenting upon the above cases, says that vomiting due to version or flexion of the uterus is removed by dilatation. He says he has accomplished the same thing by elevating the womb. Dr. Sinclair had had a case in Charlestown, of a woman who had been vomiting from the sixth week to the fourth month. She was put in the knee and elbow position with good effect.

Dr. Minot reported a case in which a sponge tent stopped the symptoms completely, much to the indignation of the woman, who had hoped to abort. In another case, seen with Dr. Clarke, Dr. Minot had applied tincture of iodine. Dr. Putnam, in consultation with Dr. Clarke, proposed removing the contents of the uterus, and a sponge tent was inserted for the purpose. The vomiting stopped. In a case seen with Dr. Buckingham, the dilatation had no effect whatever in stopping the vomiting, and the patient died.

Dr. Sinclair said he imagined there was no great danger of producing abortion by the process of dilatation spoken of. In the cases reported there is no mention of subsequent examination to see whether the os closed again.

